# MTCH2 acts as a novel inhibitory receptor to suppress germ cell mitophagy by tethering BECN1, thereby maintaining mitochondrial homeostasis and male fertility

**DOI:** 10.1038/s41419-026-09165-3

**Published:** 2026-08-18

**Authors:** Mengxing Cai, Ruifang Ren, Huiling Shu, Yanyan Yang, Longhai Dai, Yanming Dong, Xiazhou Fu, Yibin Cheng

**Affiliations:** 1 State Key Laboratory of Biocatalysis and Enzyme Engineering, Hubei Key Laboratory of Industrial Biotechnology, Hubei Collaborative Innovation Center for Green Transformation of Bio-Resources, School of Life Sciences, Hubei University, Wuhan, China; 2 State Key Laboratory of Oral & Maxillofacial Reconstruction and Regeneration, Key Laboratory of Oral Biomedicine Ministry of Education, Hubei Key Laboratory of Stomatology, School & Hospital of Stomatology, Wuhan University, Wuhan, China

**Keywords:** Mitophagy, Spermatogenesis

## Abstract

Mitochondria play a pivotal role in spermatogenesis and male fertility, in which the selective autophagic degradation of mitochondria (mitophagy) constitutes a key process. In this study, we identify mitochondrial carrier homolog 2 (MTCH2) as a novel and critical regulator of germ cell mitophagy. Localized to the mitochondrial outer membrane (OMM), MTCH2 directly interacts with the core autophagy protein BECN1, thereby inhibiting autophagic flux and specifically suppressing mitophagy. Germ cell-specific heterozygous deletion of *Mtch2* was sufficient to cause profound spermatogenic defects, including disorganized seminiferous tubules, vacuolization, mitochondrial sheath abnormalities, and significantly impaired sperm fertilization capacity. Transcriptomic profiling revealed that MTCH2 deficiency dysregulates pathways central to mitochondrial function and programmed cell death. Importantly, augmenting autophagy ameliorated mitochondrial dysfunction induced by MTCH2 deficiency, as characterized by impaired mitochondrial membrane potential, elevated mitochondrial reactive oxygen species (mtROS) production and cellular ROS levels. Collectively, our findings establish MTCH2 as an essential negative regulator of mitophagy that partners with BECN1 to maintain mitochondrial homeostasis, thereby ensuring normal spermatogenesis and male fertility.

## Introduction

Male infertility represents a significant global health challenge, accounting for approximately half of all infertility cases, primarily stemming from impaired spermatogenesis [1]. Spermatogenesis is a highly orchestrated process that occurs within the seminiferous tubules of the testis and requires continuous renewal of organelles and macromolecules to maintain cellular homeostasis. Mitochondria, being central to cellular energy metabolism, undergo substantial morphological and functional remodeling throughout spermatogenesis [2]. Accumulating evidence indicates that autophagy, a conserved lysosomal degradation pathway, serves as a critical regulatory mechanism for maintaining mitochondrial homeostasis and supporting the metabolic demands of developing germ cells [3]. Accordingly, dysfunctional mitochondria are closely linked to spermatogenic failure and male infertility, as mitochondrial defects can induce meiotic arrest, germ cell apoptosis, and testicular atrophy, ultimately leading to reduced fertility [4]. Collectively, these observations underscore the critical importance of mitochondrial quality control for male reproductive health.

Autophagy maintains cellular homeostasis by selectively degrading superfluous or damaged organelles via the autophagosome-lysosome pathway. The core mechanism involves multiple stages: phagophore nucleation, elongation, autophagosome maturation, and lysosomal fusion. A key regulator of autophagy is BECN1, which interacts with VPS34 and ATG14 to form the core PI3K complex, essential for autophagosome biogenesis [5]. The anti-apoptotic protein BCL2 inhibits autophagy by binding to and inactivating BECN1; dissociation of the BCL2-BECN1 complex liberates BECN1 to activate VPS34/PI3K signaling, thereby initiating autophagosome formation [6]. Accumulating evidence demonstrates the critical role of autophagy throughout spermatogenesis, affecting multiple germ cell types including spermatogonial stem cells, spermatogonia, spermatocytes, and spermatozoa [7]. For instance, deficiency in ATG5 disrupts sperm head morphogenesis and acrosome formation [8], whereas knockout of ATG7 impairs acrosomal vesicle fusion, resulting in aberrant sperm head morphology [9]. Moreover, excessive reactive oxygen species (ROS) perturb germ cell homeostasis by inducing DNA damage and disrupting the equilibrium between autophagy and apoptosis, consequently impairing spermatogenesis [10].

A specialized form of autophagy, mitophagy, refers to the selective degradation of damaged mitochondria and is essential for maintaining mitochondrial integrity, preventing excessive ROS accumulation, and promoting germ cell survival [11–13]. This process is primarily regulated through two major mechanisms: ubiquitin-dependent pathways (e.g., PINK1-Parkin) and receptor-mediated pathways [14]. In receptor-mediated mitophagy, specific mitochondrial outer membrane (OMM) proteins, including BNIP3, NIX, and FUNDC1, serve as receptors that engage the autophagic machinery to target dysfunctional mitochondria for degradation [15]. Despite significant progress in identifying mitophagy pathways, the key receptors that function in specific tissues, particularly in the germline, and their detailed regulatory mechanisms remain incompletely understood.

Mitochondrial carrier homolog 2 (MTCH2) is an OMM protein belonging to the SLC25A family and participates in multiple cellular physiological processes [16]. Reported functions of MTCH2 include regulating mitochondrial fusion dynamics [17], serving as a receptor for BID to activate the pro-apoptotic BAX/BAK pathway [18], and influencing cellular stress responses by modulating mitochondrial oxidation, ATP generation, and ROS levels [19, 20]. Beyond its role in apoptosis, MTCH2 participates in autophagy regulation. A recent study in adipose tissue demonstrated that MTCH2 negatively regulates autophagy through BCL2-dependent mechanisms, thereby suppressing thermogenesis [21]. Conversely, a study in *Drosophila* reported that the MTCH2 ortholog Mtch promotes mitophagy during intestinal development by regulating BNIP3 expression [22]. These seemingly opposing findings suggest that MTCH2 may exert context-dependent effects on autophagy/mitophagy. MTCH2 is also involved in ferroptosis [23, 24], highlighting its significance in maintaining metabolic and redox balance [25]. Notably, MTCH2 is highly expressed in the testis [26], suggesting a potential, though not yet defined, role in germ cell maintenance. Given that autophagy is critical for mitochondrial quality control during spermatogenesis [27], the potential role of MTCH2 in regulating germ cell mitophagy warrants further investigation.

In this study, we hypothesize that MTCH2 acts as a critical brake on mitophagy in germ cells. We identify MTCH2 as a novel regulator of mitophagy that directly interacts with BECN1 to suppress autophagic initiation. To elucidate the role of MTCH2 in male germ cell development and fertility, we generated a germ cell-specific *Mtch2*-knockout mouse model by crossing *Mtch2* ^*flox/flox*^ mice with *Stra8-Cre* transgenic mice. Phenotypic analysis revealed that germ cell-specific *Mtch2* heterozygous deletion was sufficient to cause disorganized seminiferous tubules, vacuolization, mitochondrial sheath defects, and reduced sperm fertilizing capacity. Importantly, activation of autophagy ameliorated mitochondrial dysfunction (such as impaired mitochondrial membrane potential, elevated mtROS and cellular ROS) resulting from MTCH2 deficiency. Collectively, our findings establish MTCH2 as a critical negative regulator of mitophagy that interacts with BECN1 to maintain mitochondrial homeostasis, thereby supporting normal spermatogenesis and male fertility.

## Results

### MTCH2 expression pattern in the mouse testis

**Fig. 1:**
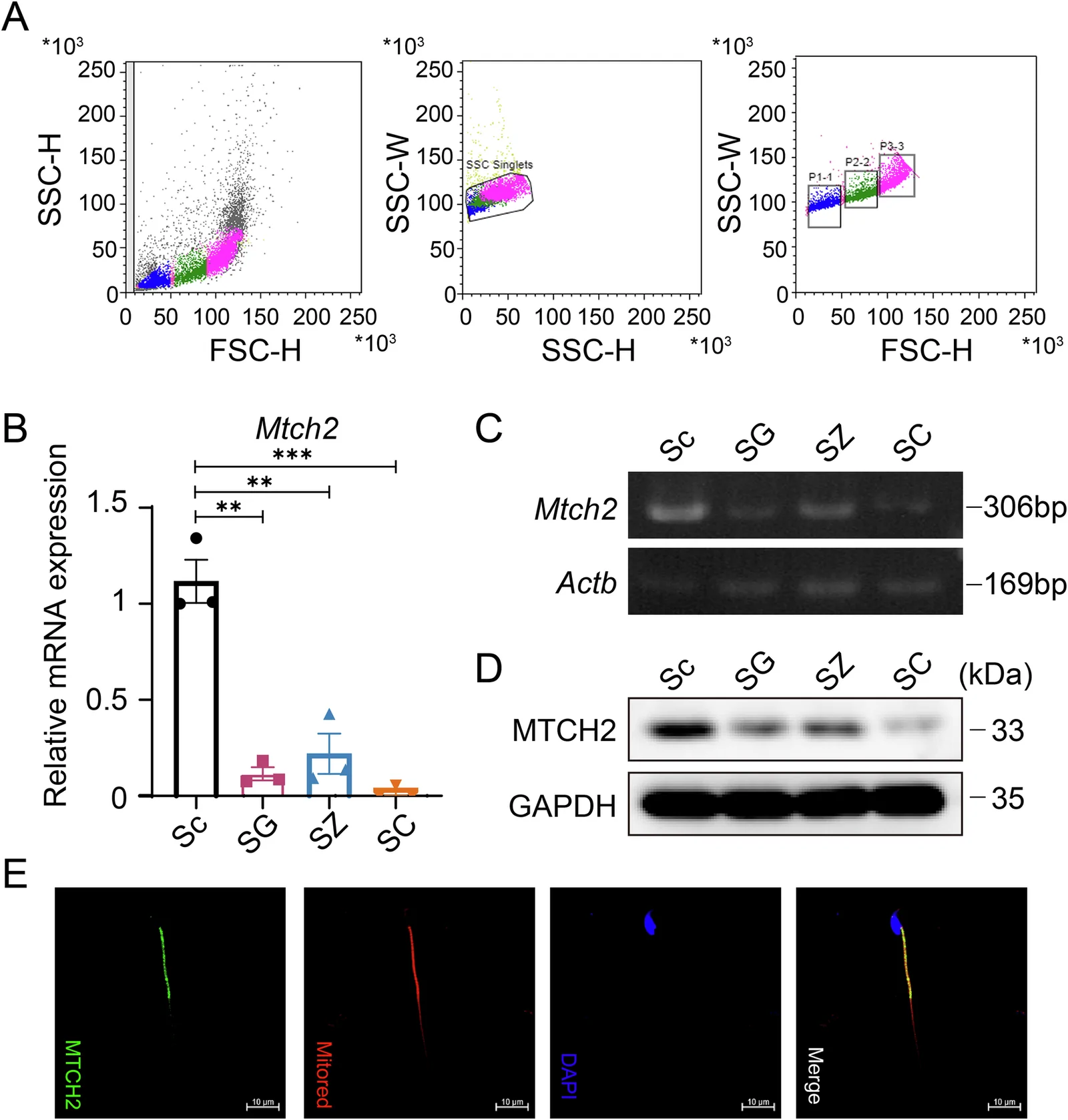
Expression profile of MTCH2 during murine testicular development. **A** Fluorescence-activated cell sorting (FACS) strategy for isolating spermatogonia (SG), spermatocytes (Sc), and spermatozoa (SZ) based on forward scatter (FSC). The gating strategy for sorting different cell populations based on cell size is shown. **B** *Mtch2* mRNA expression in sorted Sc, SG, SZ, and Sertoli cells (SC) assessed by RT-qPCR. *Actb* was used as an internal control. **p < 0.01; ***p < 0.001. **C** Representative gel images of semi-quantitative RT-PCR. *Actb* was used as an internal control. **D** Western blot analysis of MTCH2 protein in sorted testicular cell populations. GAPDH served as the loading control. **E** Subcellular localization of MTCH2 in mature sperm. Sperm were co-stained with Mito Tracker (mitochondrial matrix, red) and anti-MTCH2 antibody detected with FITC-conjugated rabbit anti-goat IgG (H + L) (green). Nuclei were counterstained with DAPI (blue). Scale bar, 20 µm.

To investigate MTCH2’s function in the germline, we first examined its expression profile using specific markers (DDX4, DAZL, SOX9) during postnatal testicular development at postnatal days 7, 14, 21 and adulthood (AD, 8 weeks). Immunofluorescence analysis revealed widespread MTCH2 expression in the cytoplasm of spermatogonia (SG), spermatocytes (Sc), spermatids (St), and Sertoli cells (SC) (Fig. S1A–C). Fluorescence-activated cell sorting (FACS) was used to isolate spermatogonia, spermatocytes and spermatozoa (SZ) (Fig. 1A); purity was validated using *Kit* (SG), *Sox9* (SC), *Sycp3* (Sc), and *Spesp1* (SZ) (Fig. S2). RT-qPCR and western blot confirmed *Mtch2* mRNA and MTCH2 protein expression in all sorted populations, with the highest levels in Sc, intermediate levels in SZ and SG, and lowest in Sertoli cells (Fig. 1B–D). This expression gradient suggests that MTCH2 may be particularly important in meiotic spermatocytes, where mitochondria undergo dynamic remodeling and require stringent quality control to ensure proper chromosome segregation and cell progression through meiosis. In mature spermatozoa, MTCH2 protein localized specifically to the mitochondrial region of the sperm midpiece, as shown by co-staining with MitoTracker (Fig. 1E). Collectively, these findings demonstrate that MTCH2 is differentially expressed across testicular cell types, with enrichment in spermatocytes, supporting its functional relevance in germ cell maintenance, particularly in contexts of high mitochondrial demand.

### MTCH2 deficiency impairs mitochondrial structure and function in spermatogonium

**Fig. 2:**
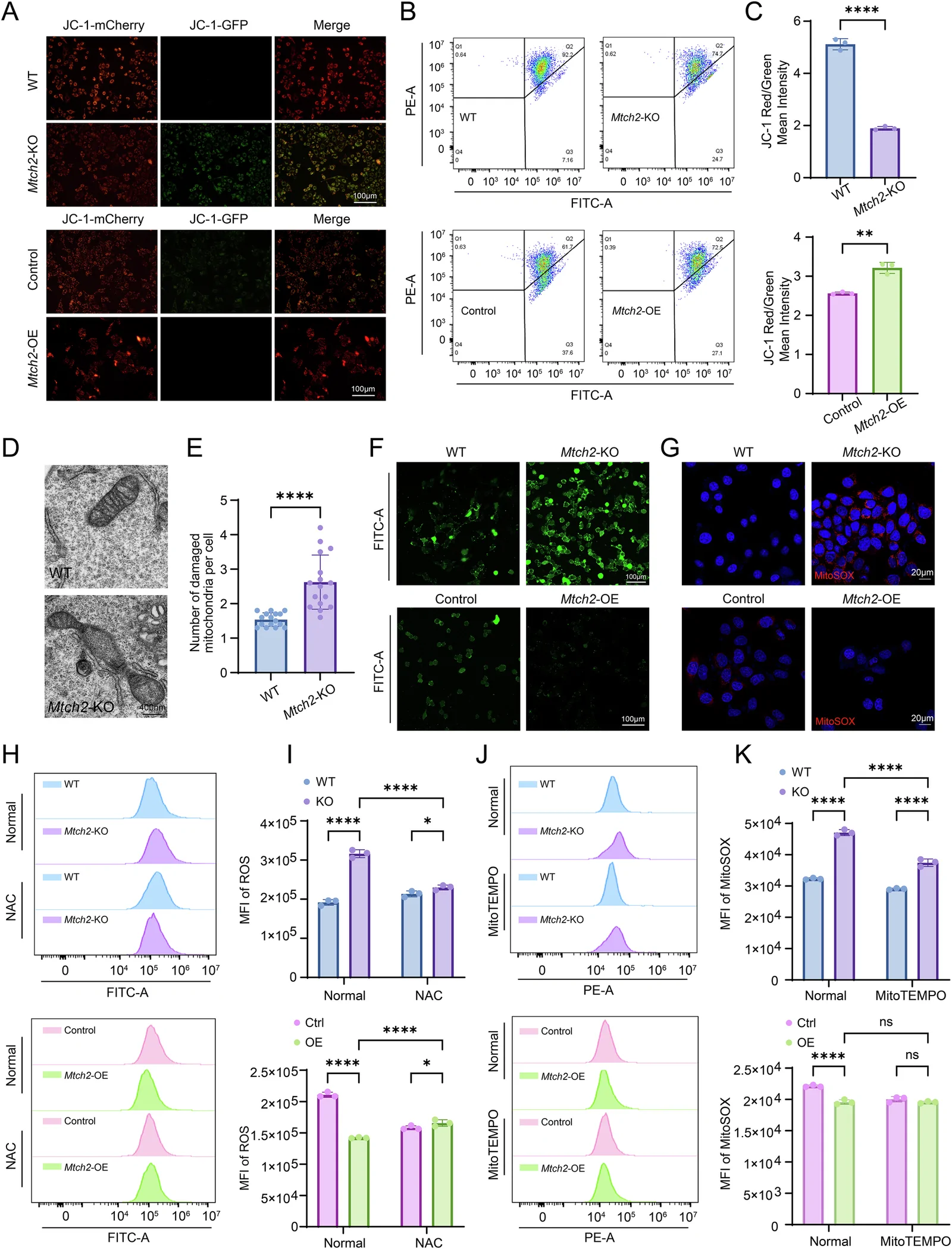
MTCH2 deficiency impairs mitochondrial function in spermatogonia. **A** Confocal microscopy of JC-1-stained cells. KO cells exhibit increased green (depolarized) and decreased red fluorescence compared to WT. Scale bar, 100 µm. **B** Flow cytometry analysis of mitochondrial membrane potential (ΔΨm) using the JC-1 assay in wild-type (WT), *Mtch2* knockout (KO), control, and *Mtch2* overexpressing (OE) GC-1 cell lines. Monomeric JC-1 (green, FITC-A) and J-aggregates (red, PE-A) fluorescence are plotted. **C** Quantification of JC-1 red/green fluorescence intensity ratio based on flow cytometry data. Data are presented as mean ± SEM. **p < 0.01, ****p < 0.0001 (*n* = 3 independent experiments). **D** Representative TEM images of mitochondria in WT and *Mtch2* KO GC-1 cells. KO cells display marked swelling, cristae fragmentation or loss, and outer membrane disruption. Scale bar, 400 nm. **E** Quantification of damaged mitochondria based on ultrastructural criteria (swelling, cristae loss ≥50%, or membrane rupture). Data are presented as mean ± SEM. *p < 0.05. **F** Confocal images of cellular ROS assessed by DCFH-DA. Scale bar, 100 µm. **G** Confocal images of mitochondrial ROS in cells assessed by MitoSOX. Scale bar, 20 µm. **H**, **I** Cellular ROS levels were assessed by DCFH-DA staining in the presence or absence of NAC (5 mM, 2 h). **H** Flow cytometry histograms. **I** Quantification of mean fluorescence intensity (MFI). Data are mean ± SD. *p < 0.05, ***p < 0.001, ****p < 0.0001 (*n* = 3 independent experiments). **J**, **K** Mitochondrial ROS levels were assessed using MitoSOX staining in the presence or absence of MitoTEMPO (10 μM, 2 h). **J** Flow cytometry histograms. **K** Quantification of mean fluorescence intensity (MFI). Data are mean ± SD. ****p < 0.0001; ns, not significant (*n* = 3 independent experiments).

To investigate the role of MTCH2 in spermatogenesis, we generated *Mtch2* knockout (KO) GC-1 spermatogonial cell lines using CRISPR/Cas9. Sequencing and off-target analysis confirmed efficient *Mtch2* ablation (Fig. S3A, B). We next assessed the impact of *Mtch2* deletion on mitochondrial function. JC-1 staining demonstrated mitochondrial depolarization in *Mtch2* KO spermatogonia, as indicated by a shift in fluorescence from red aggregates (polarized) to green monomers (depolarized) (Fig. 2A-C). Transmission electron microscopy (TEM) revealed severe mitochondrial ultrastructural defects in *Mtch2* KO GC-1 cells, including pronounced swelling, cristae fragmentation or loss, and outer membrane disruption. Quantitative analysis showed that the proportion of damaged mitochondria was significantly increased in KO cells compared to WT cells (Fig. 2D, E).

Furthermore, *Mtch2* KO significantly increased cellular ROS levels as measured by DCFH-DA (Fig. 2F). To specifically evaluate mitochondrial reactive oxygen species (mtROS) levels, we performed MitoSOX staining, which revealed a significant increase of mtROS in *Mtch2* KO cells compared to WT cells (Fig. 2G). Treatment with the ROS scavenger N-acetylcysteine (NAC) and the mitochondria-targeted antioxidant MitoTEMPO significantly reduced DCFH-DA and MitoSOX fluorescence in *Mtch2* KO cells, confirming the specificity of the detected signals. However, ROS and mtROS levels were not fully restored to wild-type levels, indicating persistent oxidative stress. This suggests that MTCH2 deficiency induces persistent ROS production due to sustained mitochondrial dysfunction, which cannot be completely reversed by antioxidant scavenging alone, and may also involve contributions from non-mitochondrial ROS sources (Fig. 2H-K). In addition, *Mtch2* KO GC-1 cells showed impaired proliferation, as determined by EdU assays (Fig. S3C).

Collectively, these data demonstrate that *Mtch2* deletion causes extensive mitochondrial damage in spermatogonia, characterized by loss of membrane potential, structural abnormalities, elevated oxidative stress, and impaired proliferation.

### MTCH2 negatively regulates autophagy and mitophagy in germ cells

**Fig. 3:**
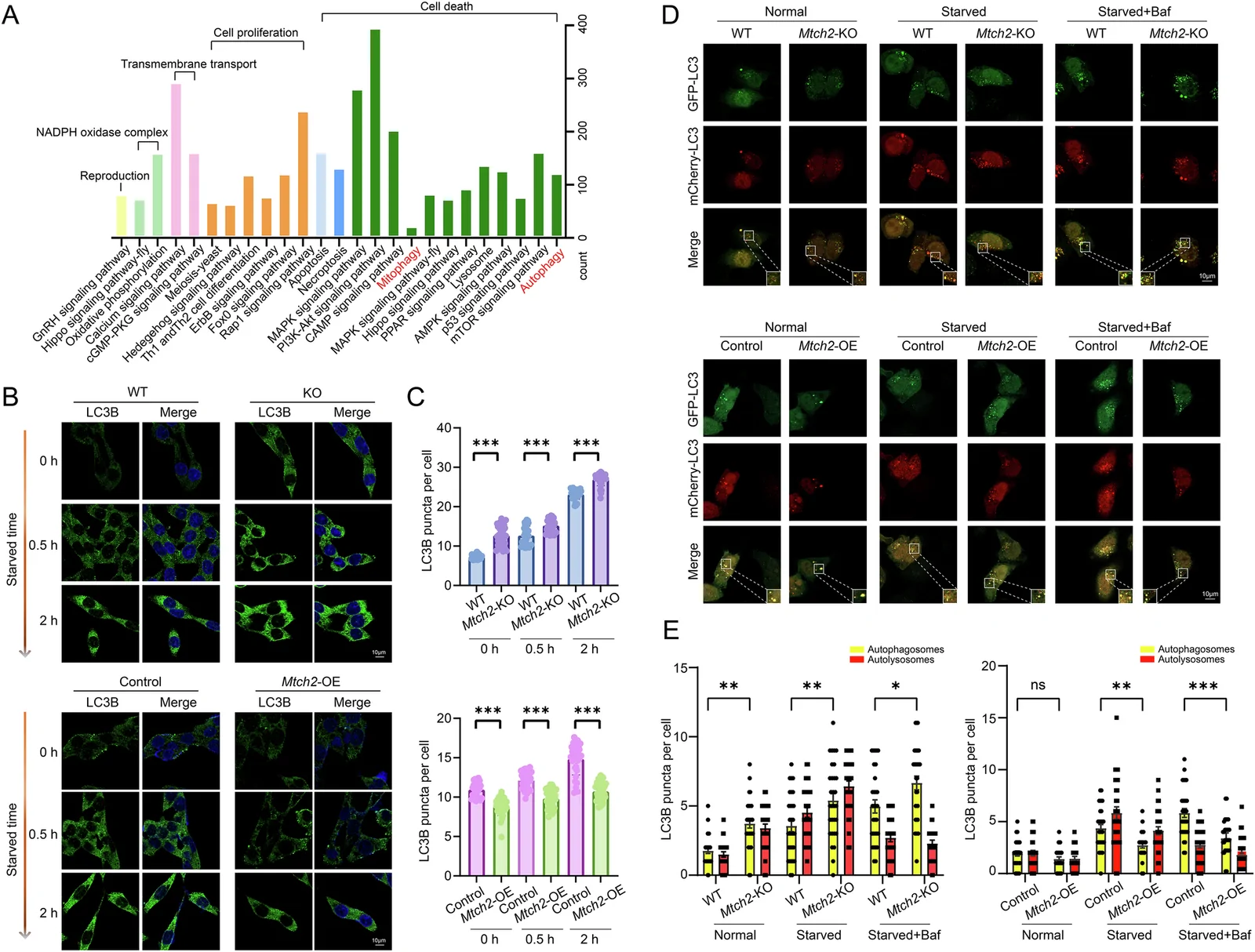
MTCH2 inhibits autophagy in GC-1 cells. **A** Gene Ontology (GO) analysis of differentially expressed genes (DEGs). The X-axis indicates the catalogs of molecular function and biological process. Y-axis on the right indicates the actual numbers of DEGs. **B** LC3B puncta formation in GC-1 cells under EBSS-induced starvation for the indicated time and analyzed by immunofluorescence with the anti-LC3B antibody (green). The nuclei were stained with DAPI reagent. Scale bar, 10 μm. **C** Quantification of LC3B puncta from (**B**). At least 30 cells were analyzed per group in each independent experiment. Data are presented as mean ± SEM. ***p < 0.001 (*n* = 3 independent experiments). **D** Representative confocal images of cells expressing the mCherry-GFP-LC3B tandem reporter under basal, starved (EBSS, 2 h), or starved with BafA1 (100 nM, 2 h) conditions. Autophagosomes appear as yellow puncta (GFP^+^/mCherry^+^), while autolysosomes appear as red-only puncta (GFP^–^/mCherry^+^). Scale bar, 10 μm. **E** Quantification of autophagosomes (yellow puncta) and autolysosomes (red-only puncta) per cell. At least 30 cells were analyzed per group in each independent experiment. Data are presented as mean ± SEM. *p < 0.05, **p < 0.01, ***p < 0.001 (*n* = 3 independent experiments).

Having established that MTCH2 deficiency leads to mitochondrial dysfunction, we next asked whether MTCH2 regulates autophagy, a key pathway for mitochondrial quality control. This question was further prompted by two lines of evidence. First, given the differential expression of MTCH2 across testicular cell types (Fig. 1D), we examined basal autophagy levels in the same sorted populations. Western blot analysis of LC3B and p62 revealed that Sertoli cells and spermatogonia, which express low levels of MTCH2, exhibited high autophagic activity, whereas spermatocytes, which express the highest MTCH2, showed the lowest autophagic activity; spermatozoa displayed an intermediate level of autophagy (Fig. S3D). This inverse correlation between MTCH2 expression and autophagic flux suggested that MTCH2 may function as a negative regulator of autophagy in germ cells. Second, transcriptomic profiling of WT and *Mtch2* KO GC-1 cells identified 4085 differentially expressed genes (DEGs) (|fold change| > 1.2, FDR < 0.05) (Fig. S3E, F). Notably, GO and KEGG analyses revealed enrichment of DEGs in pathways related to mitochondrial function and cell death, with GO terms specifically associated with mitophagy and autophagy (Fig. 3A and S3G, H). In *Mtch2* KO cells, anti-autophagy genes (e.g., Sqstm1) were downregulated, while pro-autophagy genes were upregulated (Fig. S4A–C). Together, these observations led us to directly examine the impact of MTCH2 on autophagic activity.

We first examined the effect of MTCH2 on autophagosome formation in GC-1 and HeLa cells. Under starvation conditions, immunofluorescence analysis of LC3B puncta showed accelerated autophagosome induction in *Mtch2* KO cells compared to WT cells. Conversely, *Mtch2* overexpression (OE) in WT cells decreased LC3B puncta formation following starvation (Fig. 3B, C and S5A, B). Furthermore, MTCH2 exhibited significant colocalization with LC3B in both GC-1 cells following starvation and spermatozoa (Fig. S5C, D). Using the mCherry-GFP-LC3B tandem reporter to monitor autophagic flux, we found that *Mtch2* KO significantly increased autophagosomes (yellow puncta) under starvation, while *Mtch2* OE reduced them. Treatment with bafilomycin A1 (BafA1), which blocks autophagosome-lysosome fusion, led to a marked accumulation of autophagosomes in all groups; notably, the BafA1-induced accumulation was more pronounced in *Mtch2* KO cells, indicating increased autophagic flux via enhanced autophagosome formation, rather than via a block in degradation (Fig. 3D, E). Consistently, western blot analysis showed elevated LC3B-II levels and decreased p62 protein abundance in *Mtch2* KO cells, indicative of enhanced autophagic flux. Combined starvation and BafA1 treatment amplified LC3B-II accumulation in KO cells (Fig. S6A). Conversely, *Mtch2* OE exhibited the opposite effects on LC3B-II and p62 levels (Fig. S6B). These data demonstrate that MTCH2 negatively regulates autophagosome formation and autophagic flux.

**Fig. 4:**
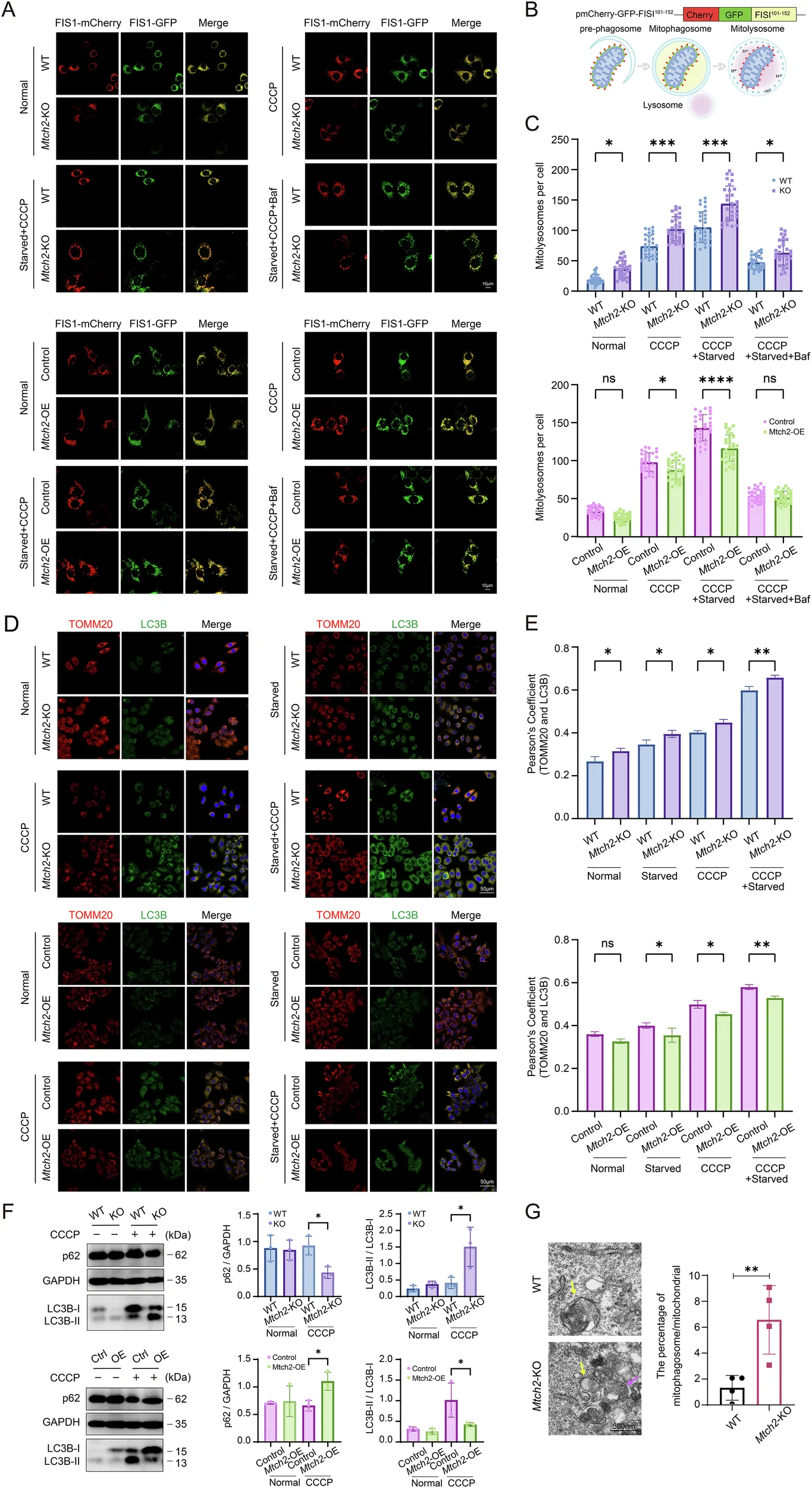
MitoQC-based analysis. **A** Fluorescent microscopy of the pmCherry-GFP-FIS1^101-152^ tandem reporter in wild-type (WT), *Mtch2* knockout (KO), Control and *Mtch2* overexpressing (OE) GC-1 cell lines. The cells were transfected with pmCherry-GFP-FIS1^101-152^ and treated under basal, CCCP (10 μM, 2 h), CCCP combined with starvation (EBSS, 2 h), and CCCP combined with starvation in the presence of BafA1 (100 nM, 2 h). Mitolysosomes: red puncta (mCherry only). Scale bar, 10 μm. **B** Schematic of MitoQC principle. pmCherry-GFP-FIS1^101-152^ tandem reporter expresses a tandem mCherry-GFP tag fused to the mitochondrial outer membrane protein FIS1; GFP fluorescence quenches in acidic lysosomes, leaving mCherry signal (red puncta) indicating mitolysosomes. **C** Quantification of mitolysosomes (red-only puncta) per cell. At least 30 cells were analyzed per group in each independent experiment. Data are presented as mean ± SEM. *p < 0.05; ***p < 0.001, ****p < 0.0001; ns, not significant (*n* = 3 independent experiments). Two-way ANOVA with Tukey’s post hoc test was used for statistical analysis. **D** Representative confocal images of LC3B (green) and TOMM20 (red) colocalization in wild-type (WT), *Mtch2* knockout (KO), Control and *Mtch2* overexpressing (OE) GC-1 cell lines under basal conditions, starvation (EBSS), CCCP (10 μM, 2 h), CCCP combined with starvation (2 h). Yellow puncta indicate LC3B-positive mitochondria (mitophagosomes). Scale bar, 50 μm. **E** Quantification of LC3B-mitochondria colocalization (Pearson’s correlation coefficient) per cell. At least 30 cells were analyzed per group. Data are presented as mean ± SEM. **p < 0.01 (*n* = 3 independent experiments). **F** Western blot analysis of LC3B-II and p62 levels in HeLa cells under indicated conditions. GAPDH was used as an internal control. Western blots were quantified for LC3B-II/LC3B-I and p62/GAPDH ratio. Data are presented as means ± SEM. *p < 0.05 (*n* = 3 independent experiments). **G** Electron microscopy showed the mitophagosome in wild-type and *Mtch2* KO GC-1 cells. Yellow arrows: autolysosomes; Purple arrows: degraded mitochondria within autolysosomes. Scale bar, 500 nm. The graphs on the right indicate statistical analysis of the percentage of mitochondria undergoing mitophagy. Data are presented as means ± SEM. **p < 0.01 (*n* = 3 independent experiments).

To directly assess mitophagy, we employed multiple complementary approaches. First, the MitoQC reporter (pmCherry-GFP-FIS1^101-152^), which targets mitochondria and reports mitophagy (mCherry-only puncta indicate mitolysosomes) was used. *Mtch2* KO significantly increased the number of mitolysosomes, whereas *Mtch2* OE decreased it, indicating enhanced mitophagy upon MTCH2 loss (Fig. 4A–C). Second, we examined the colocalization of the autophagosome marker LC3B with the mitochondrial marker TOMM20. Confocal microscopy revealed significantly increased colocalization between TOMM20 and LC3B in *Mtch2* KO cells compared to WT controls, further supporting elevated mitophagy (Fig. 4D, E). Third, we treated cells with CCCP, a classic mitophagy inducer, and assessed LC3B-II and p62 levels by western blot analysis. Under CCCP treatment, *Mtch2* KO cells showed a further accumulation in LC3B-II levels and a greater reduction in p62 compared to WT cells, while *Mtch2* OE attenuated these changes (Fig. 4F). Finally, transmission electron microscopy (TEM) of *Mtch2* KO GC-1 cells revealed damaged mitochondria sequestered within autophagosomes and autolysosomes, with a significantly higher frequency of mitophagy events compared to WT cells (Fig. 4G). Collectively, these results establish MTCH2 as a negative regulator of mitophagy in germ cells.

### MTCH2 mediates mitophagy by interacting with BECN1

**Fig. 5:**
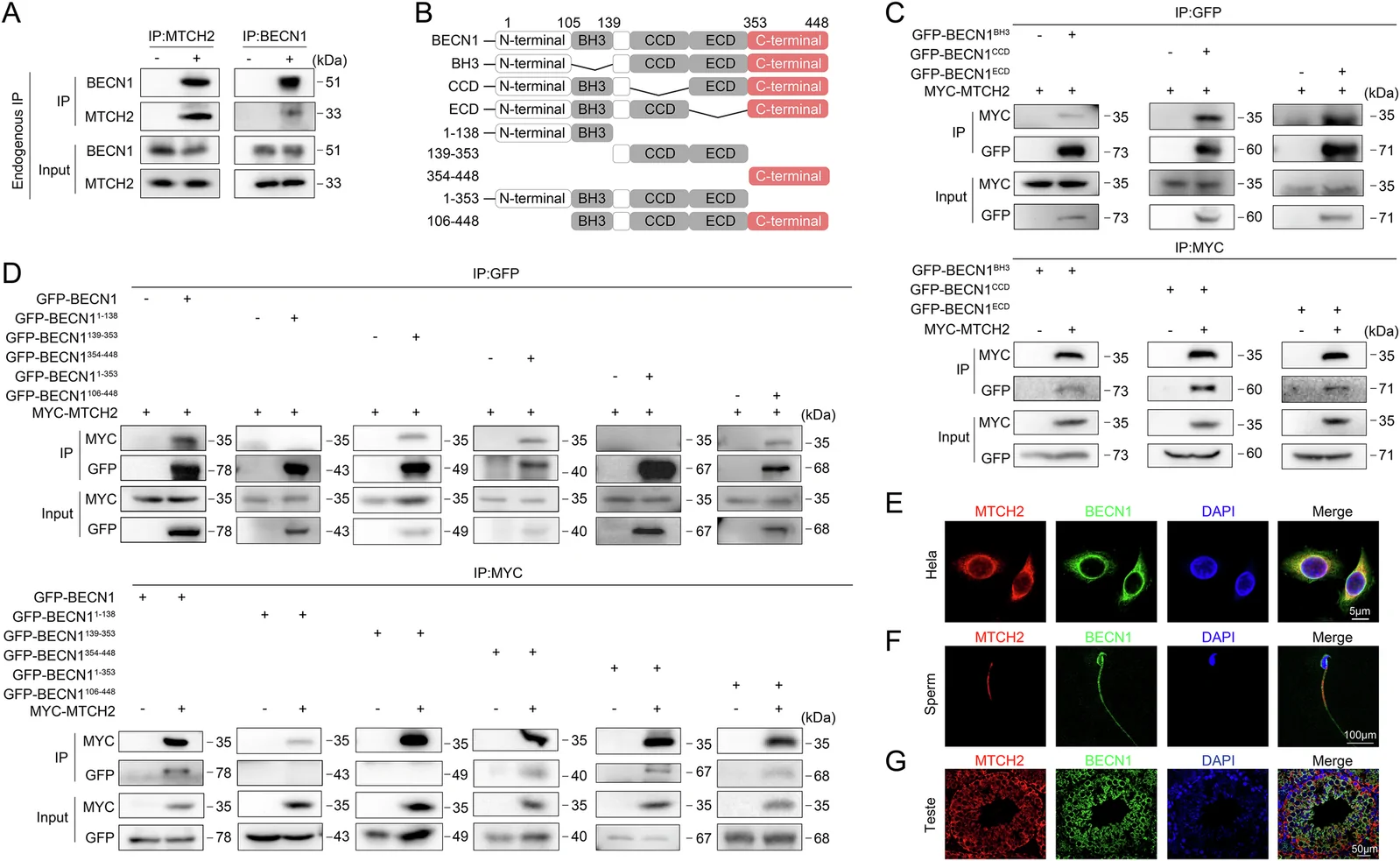
MTCH2 interacts with BECN1 via its C-terminal domain. **A** Endogenous co-immunoprecipitation (Co-IP) of MTCH2 and BECN1 from GC-1 cell lysates. The lysates were immunoprecipitated with anti-MTCH2, anti-BECN1 antibody or negative serum, respectively, followed by western blot with the anti-BECN1 or anti-MTCH2 antibody respectively. **B** Schematic of BECN1 domains and deletion mutants. The conserved domains are indicated in boxes. BH3: BCL-2 Homology 3; CCD: Coiled-Coil Domain; ECD: Evolutionarily Conserved Domain. **C** Co-IP confirming MTCH2 binding is independent of BECN1 BH3, CCD, or ECD domains using deletion mutants. MYC-MTCH2 was co-transfected with GFP-BECN1-BH3∆, GFP-BECN1-CCD∆, or GFP-BECN1-ECD∆ into 293 T cells. **D** Co-IP demonstrating MTCH2 interaction requires the BECN1 C-terminus (aa 354-448). 293T cells co-expressing MYC-MTCH2 and GFP-tagged BECN1 constructs were analyzed. **E** Confocal microscopy showing co-localization (yellow) of MTCH2 (red) and BECN1 (green) in HeLa cells. Nuclei were counterstained with DAPI (blue). Scale bar, 5 μm. **F** Co-localization of endogenous MTCH2 (red) and BECN1 (green) in sperm midpieces (mitochondria region). Nuclei were counterstained with DAPI (blue). Scale bar, 100 μm. **G** Dual immunofluorescence analysis of MTCH2 (red) and BECN1 (green) proteins in mouse testis sections. Nuclei were counterstained with DAPI (blue). Scale bar, 50 μm.

To identify MTCH2 interactors involved in autophagy initiation, we performed co-immunoprecipitation (Co-IP) analysis. We determined that BECN1, which is a key protein for autophagy initiation, could clearly interact with MTCH2 in GC-1 cell lysates (Fig. 5A). Deletion and truncation mutation analyses showed that the C-terminal domain of BECN1 is necessary and sufficient for MTCH2 binding, but not the BH3, CCD, or ECD domains (Fig. 5B–D). Furthermore, immunofluorescence demonstrated significant colocalization between MTCH2 and BECN1 in cells, spermatozoa, and testicular sections (Fig. 5E–G). These data suggest that MTCH2 may suppress mitophagy by directly tethering BECN1 at the mitochondrial outer membrane.

### Modulating autophagy rescues MTCH2 deficiency-induced mitochondrial dysfunction

**Fig. 6:**
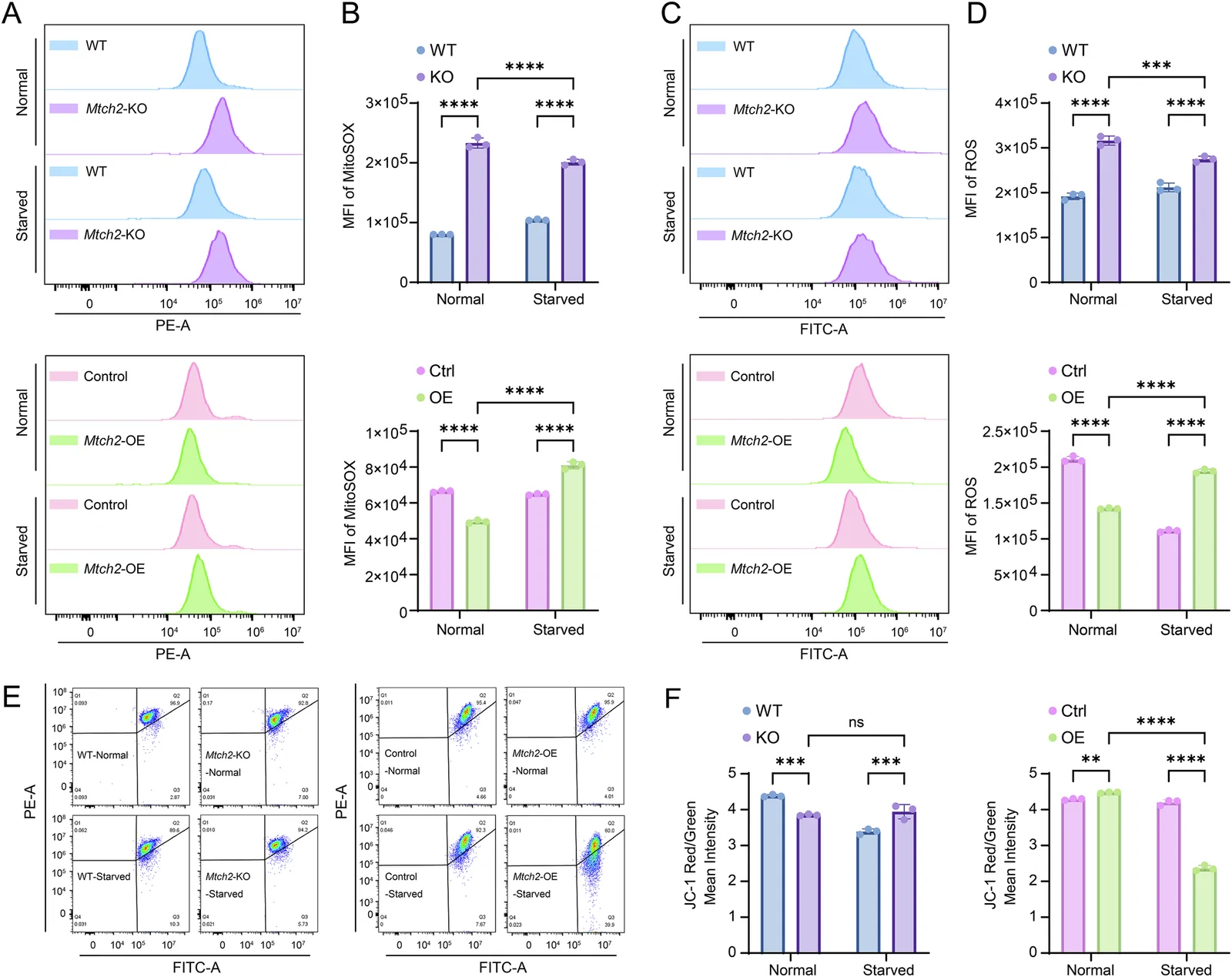
Enhancing autophagy ameliorates MTCH2 deficiency-induced mitochondrial dysfunction. **A**, **B** Mitochondrial ROS levels were assessed using MitoSOX staining under basal, starved (EBSS, 2 h) conditions. **A** Flow cytometry histograms. **B** Quantification of mean fluorescence intensity (MFI). Data are mean ± SD. ***p < 0.001, ****p < 0.0001 (*n* = 3 independent experiments). **C**, **D** Cellular ROS levels were assessed by DCFH-DA staining under basal and starved (EBSS, 2 h) conditions. **C** Flow cytometry histograms. **D** Quantification of mean fluorescence intensity (MFI). Data are mean ± SD. *p < 0.05, ***p < 0.001, ****p < 0.0001 (*n* = 3 independent experiments). **E** JC-1 flow cytometry analysis of Δψ_m_ under basal and EBSS starvation (2 h) conditions. The x-axis represents FITC-A (green fluorescence, monomeric JC-1), and the y-axis represents PE-A (red fluorescence, J-aggregates). **F** Quantification of the JC-1 red/green fluorescence intensity ratio based on flow cytometry data in (**E**). Data are presented as mean ± SEM. *p < 0.05; **p < 0.01; ***p < 0.001; ****p < 0.0001, ns, not significant (*n* = 3 independent experiments).

Given that MTCH2 deficiency causes mitochondrial dysfunction and enhances mitophagy, we tested whether modulating autophagic activity could alter mitochondrial homeostasis. Enhancing autophagy (e.g., via starvation) attenuated the elevated mtROS levels (Fig. 6A, B), ROS levels (Fig. 6C, D), and mitochondrial depolarization (Fig. 6E, F) caused by *Mtch2* deletion. Conversely, in *Mtch2*-overexpressing cells, starvation failed to lower ROS and instead caused a modest elevation, further supporting the role of MTCH2 as a negative regulator of autophagy. These results indicate that mitochondrial dysfunction in MTCH2-deficient cells can be partially rescued by further enhancing autophagy, suggesting that the elevated mitophagy upon MTCH2 loss may represent a compensatory protective response.

### Germ cell-specific heterozygous deletion of *Mtch2* impairs spermatogenesis and sperm function

**Fig. 7:**
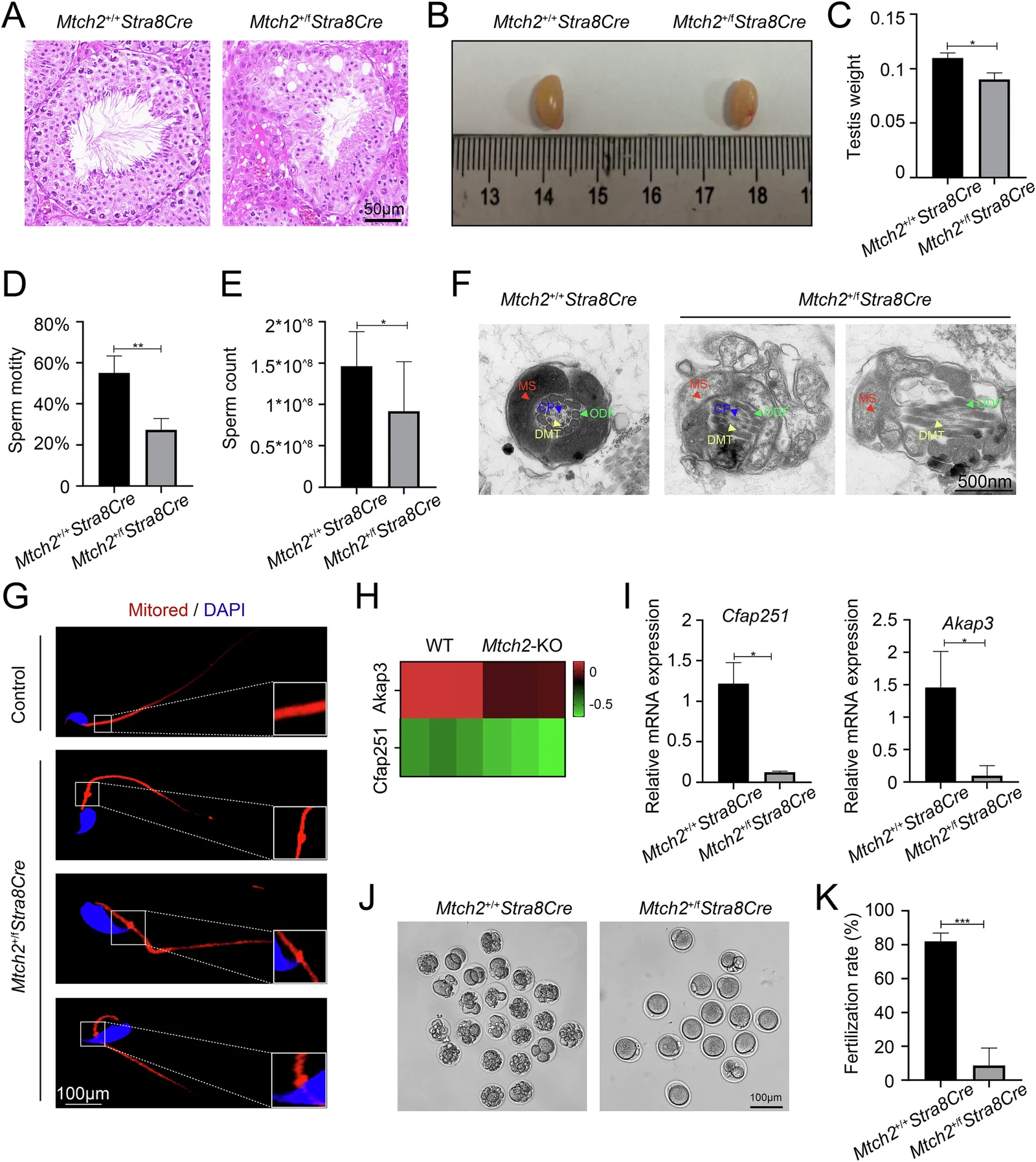
Germ cell-specific heterozygous deletion of *Mtch2* was sufficient to impair spermatogenesis and sperm function. **A** H&E staining of testis sections from adult control and heterozygous mice. Scale bar, 50 μm. **B** Photos of WT and heterozygous testes. **C** Average testis weight in control and heterozygous mice. *p < 0.05 (*N* = 3). Average percentages of motile spermatozoa (**D**) and epididymal sperm count (**E**) from WT and heterozygous mice. (*N* = 3). All data are presented as mean ± SEM and analyzed by unpaired 2-tailed Student’s *t* test. *p < 0.05, **p < 0.01. **F** TEM of sperm mid-piece cross-sections. MS (red arrow), CP (blue arrow), DMT (yellow arrow) and arrangement of ODF (green arrow). MS, mitochondrial sheath; CP, central pair; DMT, doublets of microtubules; ODF, outer dense fibers. Scale bar, 500 nm. **G** Staining of mitochondria (red) in sperm from control and heterozygous mice. Scale bar, 100 μm. **H** Heatmap of mitochondrial sheath-associated DEGs from spermatogonial transcriptomes. **I** RT-qPCR validation of *Cfap251* and *Akap3* downregulation in heterozygous testes. Data: mean ± SEM (*n* = 3), *p < 0.05. **J** Representative images of fertilized oocytes from IVF assays. Compromised in vitro fertilization competence in heterozygous sperm. Scale bar, 100 μm. **K** Quantification of fertilization rates in the control and heterozygous groups. (*N* = 3). All data are presented as mean ± SEM and analyzed using Student’s *t* test. ***p < 0.001.

To define the in vivo role of *Mtch*2 in spermatogenesis, we generated a germ cell-specific *Mtch2* conditional knockout (cKO) mouse model by crossing *Mtch2*^*flox/flox*^ mice with *Stra8-Cre* transgenic mice (Fig. S7A). Due to the impaired fertility of heterozygous mice, which made it difficult to obtain a sufficient number of appropriate homozygous mutants for experimentation, we utilized heterozygous mice for our subsequent investigations and investigated potential gene dosage effects in *Mtch2* heterozygous mice. Western blot analysis confirmed reduced MTCH2 protein levels in heterozygous testes (Fig. S7B). RT-qPCR revealed a significant reduction in *Mtch2* mRNA levels in both testes and spermatozoa of heterozygous mice (Fig. S7C). Histological analysis of testis sections (H&E staining) revealed defects in heterozygous mice, including disrupted seminiferous tubule architecture and prominent vacuolization (Fig. 7A). Consistent with the histological abnormalities, heterozygous mice exhibited significantly reduced testis size and weight compared to wild-type (WT) littermate controls (Fig. 7B, C).

Spermatozoa isolated from the cauda epididymis of heterozygous mice showed significantly reduced sperm motility and sperm count (Fig. 7D, E). We also analyzed the ultrastructure of heterozygous spermatozoa from the cauda epididymis using transmission electron microscopy (TEM), which displayed disorganized flagella including misaligned mitochondrial sheaths (Fig. 7F). Morphological assessment revealed a higher incidence of sperm tail abnormalities in heterozygous mice (Fig. 7G). To explore the molecular basis of the structural defects observed in heterozygous sperm, we analyzed the transcriptome of *Mtch2* KO GC-1 cells. This analysis revealed significant downregulation of genes associated with mitochondrial sheath formation and flagellar assembly, including *Cfap251* and *Akap3* (Fig. 7H), suggesting that MTCH2 deficiency disrupts transcriptional programs essential for sperm tail formation. RT-qPCR revealed a significant reduction in *Cfap251* and *Akap3* mRNA levels in spermatozoa of heterozygous mice (Fig. 7I). Furthermore, in vitro fertilization (IVF) assays demonstrated severely compromised fertilization capacity of spermatozoa from heterozygous mice (Fig. 7J, K). Collectively, these results establish that even partial loss of *Mtch2* in germ cells disrupts spermatogenesis, impairs sperm structure and function, and leads to male infertility.

### Autophagy is upregulated, and mitochondrial function is impaired in heterozygous *Mtch2*-deletion mice

**Fig. 8:**
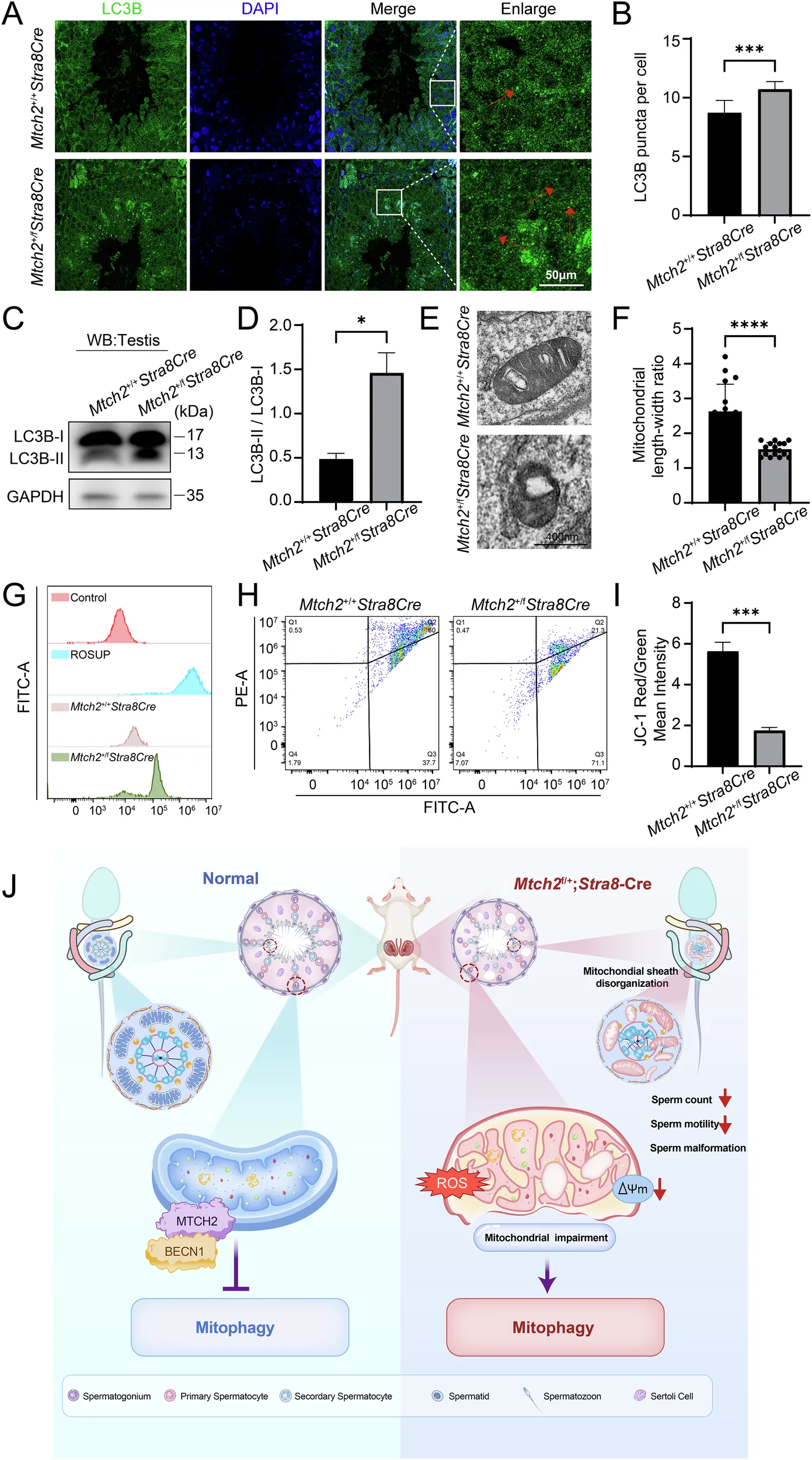
Autophagic dysregulation and mitochondrial impairment in heterozygous *Mtch2* mice. **A** LC3B immunofluorescence in heterozygous and wild-type testis sections. LC3B puncta were mainly located in the cytoplasm in both heterozygous and wild-type mice (red arrows). The nuclei were stained with DAPI reagent. The enlarged images correspond to the boxed regions. Scale bar, 50 μm. **B** Statistical analysis of LC3B puncta per cell using Student’s *t* test. ***p < 0.001. **C** Western blot analysis showed that LC3B-II was upregulated in the heterozygous testis. GAPDH was used as an internal control. **D** Quantification of LC3B-II/LC3B-I ratio in testis. The results were representative of 3 independent experiments and are represented as means ± SEM. The data were analyzed using Student’s *t* test. *p < 0.05. **E** Representative TEM images of spermatogonia in testis sections from WT and heterozygous mice. Scale bar, 500 nm. **F** Quantification of mitochondrial length/width ratio in spermatogonia from (**E**). Data are shown as mean ± SEM and analyzed using Student’s *t* test. ****p < 0.0001. **G** Flow cytometry analysis of ROS levels in control and heterozygous sperm using DCFH-DA. **H** Flow cytometry analysis of mitochondrial membrane potential using the JC-1 assay. **I** Quantification of JC-1 red/green ratios based on flow cytometry data in (**H**). Data are presented as mean ± SEM. *p < 0.05 (*n* = 3 independent experiments). **J** A model showing that MTCH2 suppresses germ cell autophagy via BECN1 to maintain mitochondrial homeostasis and male fertility.

To further investigate the molecular mechanisms underlying impaired spermiogenesis and male fertility, we detected autophagy in testes. Notably, autophagy was upregulated in heterozygous testes, evidenced by increased LC3B puncta formation and elevated LC3B-II protein levels compared to WT testes (Fig. 8A-D). TEM analysis further revealed impaired mitochondrial ultrastructure in heterozygous testes. Spermatogonia from heterozygous mice exhibited shortened mitochondria with frequent vacuolization and loss of cristae, indicative of severe mitochondrial abnormalities (Fig. 8E, F). Assessment of sperm mitochondrial function in heterozygous mice showed significantly elevated ROS levels and mitochondrial depolarization compared to WT spermatozoa (Fig. 8G–I). These findings indicate that partial loss of *Mtch2* alters autophagic activity in germ cells, compromises mitochondrial integrity and function in the testis and sperm, and contributes to the observed reproductive defects.

## Discussion

Mitochondrial homeostasis is indispensable for male fertility, underpinning critical processes such as spermatogenesis, sperm motility, and energy metabolism. Accordingly, mitochondrial dysfunction represents a major cause of spermatogenic failure and infertility [28]. During spermatogenesis, mitochondria undergo substantial morphological and functional remodeling, evolving from dispersed networks in spermatogonia to the tightly packed mitochondrial sheath in sperm tails. This process is accompanied by substantial ROS production [29], thereby necessitating robust quality control mechanisms—including antioxidant defenses, DNA repair systems, and selective degradation pathways—to maintain organelle integrity [30].

As an integral mitochondrial outer membrane (OMM) protein, MTCH2 has recently emerged as a multifaceted regulator of mitochondrial physiology [31]. Our study defines a critical role for MTCH2 in male germ cell development. We demonstrate that germ cell-specific deletion of *Mtch2* severely impairs mitochondrial function, manifesting as depolarization, pronounced swelling, cristae disorganization, and elevated cellular ROS, and mitochondrial ROS levels in spermatogonia. These cellular defects culminate in profound phenotypic outcomes, including significant testicular atrophy, reduced testis size and weight, disorganized seminiferous tubules, and prominent vacuolization. Our data, consistent with prior reports linking MTCH2 deficiency to aberrant mitochondrial respiration and energy depletion [20], underscore that precise regulation of MTCH2 expression is essential for mitochondrial health and functional spermatogenesis, as both its overexpression [32] and germline-specific deletion [33] result in subfertility or complete infertility.

The structural integrity of the sperm mitochondrial sheath, which is compromised in the heterozygous model, is paramount for fertility. The flagellum’s mitochondrial sheath supplies energy for motility and sperm-egg fusion. Our transcriptomic analysis of MTCH2-deficient spermatogonia revealed significant downregulation of genes associated with mitochondrial sheath and flagellar assembly, providing a molecular basis for the observed structural and functional deficits. This aligns with studies showing that deficiencies in other sheath-associated proteins like AKAP3, AKAP4 [34], CFAP45 [35], CFAP251 [36, 37]or DNAH17 [38] similarly cause flagellar abnormalities and male infertility. Thus, MTCH2 joins a critical group of factors essential for maintaining mitochondrial sheath integrity and functional competence in sperm. This is directly evidenced in our heterozygous mouse model by reduced sperm count, impaired motility, and a severely compromised fertilization capacity.

The most significant finding of our study is the identification of MTCH2 as a novel negative regulator of mitophagy in germ cells. Mitophagy represents the core pathway for mitochondrial quality control, selectively eliminating damaged or superfluous mitochondria to maintain organelle homeostasis. This process is essential for regulating diverse cellular functions and cell fate decisions, and is particularly critical for spermatogenesis [39]. It can be initiated by various stressors, including nutrient deprivation, hypoxia, and direct mitochondrial damage [40]. Our data support a model in which MTCH2 functions as a specialized inhibitory receptor that tethers the key autophagy initiator BECN1 at the OMM to prevent inappropriate autophagosome formation in germ cells. This function stands in direct contrast to canonical mitophagy receptors (e.g., BNIP3, NIX, FUNDC1), which typically recruit autophagy machinery to promote the degradation of damaged mitochondria [41–44]. While these receptors act as “eat-me” signals, our findings position MTCH2 as a “don’t-eat-me”-like signal on healthy mitochondria, representing a novel paradigm in receptor-mediated mitophagy control. This receptor diversity underscores the complexity and potential cell/tissue specificity of mitophagy initiation mechanisms.

A key observation supporting this negative regulatory role is the inverse correlation between MTCH2 expression levels and basal autophagic activity across different testicular cell types. Using sorted testicular cell populations, we found that Sertoli cells and spermatogonia (low MTCH2) exhibit high LC3B-II and low p62 levels, whereas spermatocytes and spermatozoa (high MTCH2) show the opposite pattern. This expression gradient is consistent with a recent in vivo autophagy mapping study which demonstrated dynamic autophagic flux across spermatogenesis stages using a tfLC3-KI reporter [27]. Thus, we propose that MTCH2 serves as a “rheostat” that fine-tunes mitophagy permissiveness according to the physiological demands of each developmental stage: high MTCH2 in spermatocytes protects mitochondria during active proliferation and meiotic progression, while lower MTCH2 in later stages permits necessary mitochondrial remodeling.

Our conclusion that MTCH2 acts as a negative regulator of mitophagy in germ cells appears to differ from a recent study in *Drosophila* [22], which reported that the *Drosophila* ortholog of MTCH2, Mtch, promotes mitophagy during intestinal development, acting in a pathway with Vps13D, PINK1, Parkin, and the mitophagy receptor BNIP3. Several factors may explain this apparent discrepancy. Cell type- and tissue-specific contexts likely determine distinct functions of MTCH2. In *Drosophila* enterocytes, developmentally programmed mitophagy is essential for intestinal remodeling, requiring active mitochondrial clearance. In contrast, in mouse germ cells, inappropriate mitophagy during early spermatogenesis could compromise the integrity of the mitochondrial pool needed for subsequent flagellum formation. Moreover, the molecular partners may differ: we demonstrate direct binding to BECN1, whereas the *Drosophila* study implicates a pathway involving BNIP3. Mechanistically, BECN1 functions at autophagosome initiation via the class III PI3K complex. Our finding that MTCH2 interacts with BECN1, and that MTCH2 loss accelerates LC3B puncta formation and autophagic flux (mCherry-GFP-LC3B and BafA1 assays), supports MTCH2 acting as an inhibitory brake at the initiation stage. Finally, our conclusion that MTCH2 negatively regulates autophagy is consistent with a recent study in adipose tissue [21], which showed that MTCH2 suppresses thermogenesis by negatively regulating autophagy via a Bcl-2-dependent mechanism. Collectively, these observations highlight MTCH2 as a versatile node integrating mitochondrial status with autophagic activity across multiple tissues, with its precise output—promotion or suppression—determined by cell type, metabolic state, and developmental stage.

As previously discussed, accumulated evidence demonstrates the indispensable role of autophagy across the different stages of spermatogenesis. In this study, we identify MTCH2 as a novel regulator of mitophagy in germ cells (Fig. 8J). As an integral OMM protein, MTCH2 acts as a critical brake on autophagic initiation, and our data demonstrate that it suppresses overall autophagic flux under stress conditions. Functional analyses revealed that MTCH2 deficiency induces pronounced mitochondrial damage and significantly elevates mitophagy. Importantly, restoration of normal autophagic activity ameliorated mitochondrial dysfunction in MTCH2-deficient cells, rescuing the associated elevations in mitochondrial ROS, total ROS and loss of membrane potential. Mechanistically, we establish that MTCH2 directly interacts with the core autophagy protein BECN1, specifically via its C-terminal domain, to restrain autophagic initiation under stress conditions (e.g., starvation, mitochondrial damage). This interaction positions MTCH2 as a unique selectivity factor on the OMM, directly tethering BECN1 to safeguard mitochondria from inappropriate degradation under homeostatic conditions. Notably, MTCH2’s regulatory mode diverges fundamentally from canonical autophagy receptors: rather than recruiting machinery to damaged cargo, it directly tethers the key autophagy initiator BECN1 at the mitochondrial surface, thereby limiting autophagosome formation.

In conclusion, our findings establish MTCH2 as a crucial negative regulator of germ cell mitophagy and delineate a novel mechanism that, when disrupted, leads to male infertility. By directly engaging and sequestering BECN1 at the mitochondrial surface, MTCH2 exerts precise spatiotemporal control over mitophagy initiation. This discovery expands the mechanistic repertoire of mitochondrial quality control, introducing a new paradigm of receptor-mediated mitophagy inhibition, and establishes the MTCH2-mediated pathway as vital for germ cell development and male fertility. Future studies should aim to elucidate the upstream signals that regulate the MTCH2-BECN1 interaction in response to metabolic or oxidative stress. It is tempting to speculate that this interaction may be modulated by post-translational modifications in response to metabolic or oxidative stress, or through competitive displacement by other BECN1-binding partners, akin to the regulation of the BECN1-BCL2 complex. Elucidating these regulatory inputs will be key to understanding how mitophagy is precisely tuned during the demanding process of spermatogenesis. Given that dysregulation of this pathway leads to significant reproductive pathologies, modulating MTCH2-mediated mitophagy may represent a potential therapeutic strategy for certain forms of male infertility.

## Materials and methods

### Animals

All C57BL/6 mice were obtained from the Hubei Provincial Center for Disease Control. This study was conducted in accordance with the guiding principles for the ethical care and use of animals in biomedical research.

### Antibodies and reagents

Primary Antibodies: Anti-MTCH2 (Sigma, Cat# 383AFA76); Anti-LC3B (Sanying, Cat# 14600-1-AP); Anti-BECN1 (ABclonal, Cat# A22361); Anti-GAPDH (Servicebio, Cat# AC24031001); Anti-FLAG (Sanying, Cat# 66008-4-IG); Anti-MYC (Sanying, Cat# AC250308013); Anti-DAZL (ABclonal, Cat# A13970); Anti-DDX4 (Sigma, Cat# AB270534); Anti-p62 (ABclonal, Cat# A19700); Anti-GFP (ABclonal, Cat# AE012); Anti-TOMM20 (Abiowell, Cat# AWA54820).

Secondary Antibodies: Peroxidase-AffiniPure Goat Anti-Rabbit IgG, Light Chain Specific (Jackson ImmunoResearch, Cat# A25022); Peroxidase-AffiniPure Rabbit Anti-Mouse IgG, Fcγ Fragment Specific (Jackson ImmunoResearch, Cat# SA0001-1); Goat Anti-Mouse IgG (H + L)-HRP Conjugate (Thermo Fisher Scientific, Cat# 31460); Fluorescein (FITC)-AffiniPure Goat Anti-Rabbit IgG (H + L) (OriGene, Cat# ZF-0311).

Reagents: ROS Assay Kit (Servicebio, Cat# MPC240720); Mitochondrial Membrane Potential Assay Kit (Servicebio, Cat# MPC240715); MitoSOX Red (MedChemExpress, Cat# 1003197-00-9); MitoTEMPO (MedChemExpress, Cat# 1334850-99-5); Bafilomycin A1 (MedChemExpress, Cat# HY-100558); N-acetylcysteine (Sigma, Cat# A9165); Carbonyl cyanide m-chlorophenyl hydrazone (Sigma, Cat# C2759); Click-iT^TM^ EdU kit (Thermo Fisher, Cat# C10337); DAPI (Sigma, Cat# D9542).

### Cell culture and transfection

HEK293T, GC-1, and HeLa cell lines were obtained from the Chinese National Collection of Authenticated Cell Cultures. Cells were maintained in high-glucose Dulbecco’s modified Eagle Medium (HyClone, Cat# SH30022.01B) supplemented with 12% (v/v) fetal bovine serum (PAN-Biotech, Cat# P30-330250) at 37°C in a humidified incubator with 5% CO₂. For transfection assays, cells were seeded into culture plates or onto glass coverslips. Transfection was performed using polyethylenimine (MedChemExpress, Cat# HY-W250110) according to standard protocols. To induce autophagy via nutrient deprivation, cells were incubated in Earle’s Balanced Salt Solution (HyClone, Cat# SH30029.02) for the specified durations. All cell lines were authenticated by short tandem repeat (STR) profiling and confirmed to be free of mycoplasma contamination.

### Western blot and co-immunoprecipitation (Co-IP)

Western blot was conducted following standard protocols. Briefly, cellular protein extracts were resolved by electrophoresis on appropriate percentage SDS-polyacrylamide gels and subsequently electrotransferred onto 0.2 μm polyvinylidene difluoride membranes (Sigma, Cat# ISEQ00010). Membranes were incubated with primary antibodies overnight at 4 °C. Following incubation, membranes were washed twice with Tris-buffered saline containing 0.1% Tween 20, incubated with appropriate horseradish peroxidase (HRP)-conjugated secondary antibodies for 1 h at room temperature, and washed thrice with TBST. Protein bands were visualized using enhanced chemiluminescence (ECL) substrate (Sigma, Cat# WBKLS0100). For Co-IP analysis of protein interactions, HEK293T cells were co-transfected with the indicated plasmids. Endogenous immunoprecipitation was performed in GC-1 cells using anti-MTCH2 or anti-BECN1 antibodies, with normal rabbit or mouse IgG used as species-matched negative controls. After 48 h, cells were lysed in lysis buffer (50 mM Tris-HCl pH 8.0, 0.15 M NaCl, 1 mM EGTA, 0.5% NP-40). Subsequent immunoprecipitation and washing steps were performed according to standard procedures.

### Immunofluorescence (IF) analysis

Testicular tissues were fixed, processed, and embedded in paraffin. GC-1 and HeLa cells were grown on glass coverslips. Tissue sections and coverslip-adherent cells were fixed with 4% paraformaldehyde for 20 min at room temperature, permeabilized with either 1% or 0.1% Triton X-100 in PBS for 30 min, and then incubated with specific primary antibodies overnight at 4 °C. Subsequently, samples were incubated with fluorophore-conjugated secondary antibodies at room temperature for 1 h. Fluorescent images were acquired using a confocal laser scanning microscope (LSM 980, Zeiss, Germany). For LC3B puncta quantification, at least 30 cells per group were analyzed in each independent experiment. The number of puncta per cell was quantified using ImageJ software.

### Autophagic flux assay

Autophagic flux was assessed using HeLa cell lines (wild-type, *Mtch2*-knockout, Control and *Mtch2*-overexpressing) cultured on coverslips and transiently transfected with the pmCherry-GFP-LC3B tandem reporter plasmid. After 24 h under standard culture conditions, cells were subjected to one of the following treatments for 2 h: normal growth medium (control), EBSS medium (starvation), or EBSS medium supplemented with 100 nM Bafilomycin A1 (BafA1; to block autophagosome-lysosome fusion). Immunofluorescence staining and imaging were then performed as described above using the confocal microscope (LSM 980). For quantification, at least 30 cells per group were analyzed in each independent experiment. Autophagosomes were defined as puncta with both GFP and mCherry signals (yellow), while autolysosomes were defined as puncta with mCherry signal only (red). Quantification was performed using ImageJ software with the puncta analyzer plugin.

### MitoQC assay

GC-1 cells were transfected with the pmCherry-GFP-FIS1^101-152^ reporter plasmid. After 24 h, cells were exposed to the following conditions: basal; CCCP (10 μM, 2 h); CCCP combined with EBSS starvation (2 h); and CCCP combined with EBSS starvation in the presence of BafA1 (100 nM, 2 h). Cells were fixed and imaged using a Zeiss LSM 980 confocal microscope. For quantification, at least 30 cells per group were analyzed in each independent experiment. Mitolysosomes were quantified as mCherry-only puncta per cell using ImageJ.

### LC3B and mitochondria colocalization

GC-1 cells were fixed and immunostained with antibodies against the mitochondrial outer membrane protein TOMM20 and LC3B, followed by incubation with Alexa Fluor 488-conjugated secondary antibodies. Confocal images were acquired, and colocalization was quantified using Pearson’s correlation coefficient (ImageJ, JACoP plugin) from at least 30 cells per group.

### Flow cytometry

Testes were aseptically excised from adult male mice and placed in ice-cold PBS. The tunica albuginea was carefully removed using sterile fine forceps. The decapsulated tissue was minced in a clean dish and enzymatically digested at 37 °C for 2 h under gentle agitation in a solution containing 0.25% trypsin and 0.2% collagenase I. Digestion was terminated by adding DMEM/F12 medium supplemented with 20% FBS. The cell suspension was repeatedly pipetted to achieve dissociation, passed through a 200-mesh sieve into a fresh centrifuge tube, and centrifuged at 1000 rpm for 10 min at room temperature. The supernatant was discarded, and the cell pellet was resuspended in 2 mL of medium containing 20% FBS and penicillin-streptomycin. Cells were subsequently subjected to flow cytometry for analysis or sorting based on fluorescence intensity and light-scattering properties (forward scatter, FSC, indicative of cell size; side scatter, SSC, indicative of granularity/complexity).

For intracellular ROS detection, cells were incubated with DCFH-DA at 37 °C for 30 min in the dark. For mitochondrial ROS measurement, cells were stained with 5 μM MitoSOX Red at 37 °C for 30 min. For mitochondrial membrane potential analysis, cells were incubated with JC-1 working solution at 37 °C for 20 min according to the manufacturer’s instructions. After washing with PBS, fluorescence signals were analyzed using a CytoFLEX flow cytometer (Beckman Coulter). Mean fluorescence intensity (MFI) was quantified using FlowJo software. Quantification was performed within the gated viable cell-enriched population following standard flow cytometry analysis practices.

### RT-qPCR

RNA was extracted using an RNA extraction kit (Tiangen Biotech, Cat# 4992858) and reverse transcribed into cDNA using a reverse transcription kit (Tiangen Biotech, Cat# Y2026). Specific primers were designed, and quantitative PCR was performed using a qPCR instrument (Bio-Rad). *Actb* was used as an internal control.

### Lentivirus production, infection, and gene knockout

Lentiviral particles were produced by co-transfecting HEK293T cells with polyethylenimine (PEI). The transfection mixture included the transfer plasmid, along with the packaging plasmids pRSV-Rev (Addgene, Cat# 12253), pMD2.G (Addgene, Cat# 12259), and pCMV-VSV-G (Addgene, Cat# 8454). The supernatant was collected 48 h post-transfection and filtered. HeLa and GC-1 cells were transduced with the viral supernatant plus polybrene, followed by puromycin selection to establish stably transduced cell lines. For overexpression experiments, both the control and OE groups were generated using the same lentiviral vector backbone containing puromycin resistance and FLAG-tag elements. Primer sequences for construct validation or target amplification are provided in the relevant Supplementary Table 1.

### Transmission electron microscopy (TEM)

GC-1 Cells: Cells were pelleted and fixed in 2.5% glutaraldehyde in 0.1 M phosphate buffer (pH 7.4) at 4 °C for a minimum of 2 h.

Sperm: Caudal epididymides were dissected and placed in pre-warmed (37°C) phosphate-buffered saline for 10 min to facilitate sperm release. The sperm-containing suspension was collected, centrifuged at 900 rpm for 3 min, and the pellet was resuspended and fixed in primary fixative.

Mouse Testis Tissue: The tunica albuginea was removed, and small pieces of seminiferous tubule-containing testicular tissue were immediately immersed in primary fixative.

All fixed samples underwent standard TEM processing procedures, including post-fixation in 1% osmium tetroxide, dehydration through an ethanol series, infiltration with epoxy resin embedding, polymerization, ultrathin sectioning (60-80 nm), and staining with uranyl acetate and lead citrate. Processed samples were imaged using a transmission electron microscope.

For quantitative analysis of mitochondrial ultrastructure in cultured GC-1 cells, at least 15 cells per group were examined. Mitochondria were classified as “damaged” when at least two of the following criteria were met: (i) cross-sectional area >1.5× the mean area of WT mitochondria, (ii) loss of >50% of cristae structures, or (iii) visible outer membrane rupture. Mitochondrial aspect ratio (length/width) was measured using ImageJ software. Mitochondrial vacuolization was defined as the presence of substantial electron-lucent areas within the mitochondrial matrix occupying >20% of the organelle cross-sectional area.

### EdU Cell Proliferation Assay

Cells were seeded into 24-well plates and cultured for 24 h. Subsequently, cells were incubated with 10 μM EdU for 4 h and processed according to the manufacturer’s instructions. Nuclei were counterstained with DAPI. Fluorescence images were captured using a Zeiss LSM 980 confocal microscope.

### In vitro fertilization (IVF) assay

Female mice were intraperitoneally injected with pregnant mare serum gonadotropin (PMSG). 48 h later, mice were injected with human chorionic gonadotropin (hCG). At the time of IVF preparation (14–16 h after hCG injection), male mice were euthanized by cervical dislocation, and the caudal epididymides were isolated. Using fine forceps, sperm were expressed from a small incision made in the caudal epididymis. Sperm were placed in sperm capacitation medium and incubated at 37°C for 1 h. Oviducts from superovulated females were isolated and placed in M2 medium (under mineral oil). Oviducts were held under a microscope, the ampulla was torn open with a needle, and cumulus-oocyte complexes (COCs) were released. COCs were treated with 0.1% hyaluronidase in M2 medium to remove cumulus cells, washed 3–4 times in M2 medium, and then transferred to fertilization medium. A defined volume of capacitated sperm suspension was added to fertilization drops containing oocytes. After 6 h of co-incubation, oocytes were examined for signs of fertilization (two-pronuclei (2PN) formation) and transferred to KSOM medium for culture. Fertilization rates (2PN formation rates) were assessed at 10 h, and cleavage rates were assessed at 24 h post-insemination.

### Hematoxylin and eosin (H&E) staining

Testes were dissected and fixed in Bouin’s solution for 24–48 h at room temperature. Fixed tissues were processed, which included dehydration through a graded ethanol series, clearing in xylene, paraffin embedding, sectioning at 5 μm thickness, and mounting on glass slides. Deparaffinized and rehydrated sections were stained with Mayer’s Hematoxylin for 3 minutes, rinsed in running tap water for 2 min, counterstained with Eosin Y for 2 min, briefly rinsed, dehydrated through graded alcohols, cleared in xylene, and mounted.

### Mouse genomic DNA extraction

Approximately 0.2–0.5 cm of mouse tail tip was excised and placed in a 1.5 ml Eppendorf tube containing 150 µL of genomic DNA lysis buffer. 20 µL of Proteinase K was added to a final concentration of 2 mg/mL. The tube was vortexed and incubated in a water bath at 55 °C for at least 3 h. 200 µL of binding buffer (6 M GuHCl, 20 mM Tris-HCl, pH 6.6) and 100 µL of pre-chilled isopropanol were added (−20 °C). The mixture was centrifuged at 13,000 rpm for 30 s. The clarified supernatant was carefully transferred to a DNA adsorption column, followed by centrifugation at 13,000 rpm for 30 seconds, and the flow-through was discarded. The column was washed with 500 µL of Wash Buffer 1 (IR1), followed by centrifugation at 13,000 rpm for 30 s. After discarding the flow-through, 500 µL of Wash Buffer 2 (IR2) was added, followed by centrifugation at 13,000 rpm for 30 s. This washing step was repeated once. The column was centrifuged at 13,000 rpm for 2 minutes to dry the membrane. DNA was eluted with 100 µL of Elution Buffer and stored at −20 °C.

### Sperm Count

Caudal epididymides were dissected, placed in a microcentrifuge tube containing 1 mL of pre-warmed PBS (37 °C), and minced thoroughly with fine scissors. The tissue suspension was incubated at 37 °C for 10–15 min to allow sperm release. The suspension was gently pipetted to disperse sperm clusters and filtered through a 70 μm cell strainer to remove tissue debris. The filtered sperm suspension was diluted to achieve an appropriate sperm density for hemocytometer counting. Ten microliters of the diluted suspension were loaded onto both chambers of a hemocytometer. Sperm heads were counted under a microscope. The average count per large square was calculated.

### Generation of conditional knockout mouse model

Mouse Lines: A *Mtch2* conditional knockout (*Mtch2*^*flox/flox*^) mouse line on a C57BL/6 background was utilized. In this line, exons critical for *Mtch2* function (exons 2–8) are flanked by *loxP* sites. This line was obtained from Cyagen Biosciences (Suzhou) Inc. Mice were maintained under specific pathogen-free (SPF) conditions with ad libitum access to food and water on a 12 h light/dark cycle.

Breeding Strategy: Homozygous *Mtch2* ^*flox/flox*^ mice were crossed with *Stra8-IresCre* transgenic mice (expressing Cre recombinase specifically in male germ cells, initiating from spermatogonia). Germ cell-specific *Mtch2* heterozygous deletion mice (*Mtch2*^*fl/+*^*; Stra8-IresCre*) and Cre-positive littermate controls (*Mtch2*^+/+^*; Stra8-IresCre*) were generated.

Genotyping: Genomic DNA was extracted from tail biopsies. Genotyping PCR was performed using specific primer sets to detect the floxed *Mtch2* allele and the *Stra8-IresCre* transgene. PCR primer sequences are detailed in Supplementary Table 1.

### Statistical analysis

Data are presented as mean ± SEM as indicated in figure legends. Comparisons between two groups were performed using an unpaired two-tailed Student’s *t* test. Multiple group comparisons were analyzed by one-way or two-way ANOVA followed by Tukey’s post hoc test. A *p* value < 0.05 was considered statistically significant. All experiments were independently repeated at least three times. All analyses were performed using GraphPad Prism 9.0 software.

## Supplementary information

Supplementary information

Figure S1

## Data Availability

All relevant data are available from the corresponding author upon reasonable request.
