# Supplementary material for: MTCH2 acts as a novel inhibitory receptor to suppress germ cell mitophagy by tethering BECN1, thereby maintaining mitochondrial homeostasis and male fertility
Source: Cell Death Dis. 2026 Aug 18;17(1):963. doi: 10.1038/s41419-026-09165-3 (PMC13760039; doi:10.1038/s41419-026-09165-3)
Supplement: Supplementary file 1 [file 41419_2026_9165_MOESM1_ESM.doc]

**Supplemental Figures and Legends**

**Figure S1**

**Figure S1. Expression profile of MTCH2 during murine testicular development.**

(A-C) Immunofluorescence co-localization of MTCH2 with germ cell and Sertoli cell markers in mouse testis sections. Markers: (A) DDX4 (spermatocytes and round spermatids, red), (B) DAZL (spermatogonia and early spermatocytes, red), (C) SOX9 (Sertoli cell nuclear marker, red). MTCH2 is shown in green. Nuclei were counterstained with DAPI (blue). Merged images show colocalization (yellow) in the cytoplasm of germ cells. Scale bar: 20 μm.

**Figure S2**

**Figure S2. Validation of cell type purity by agarose gel analysis.**

(A) Purity assessment of FACS-sorted spermatocytes (Sc), spermatogonia (SG), and spermatozoa (SZ), together with primary Sertoli cells (SC), by agarose gel analysis using cell markers: *Sycp3* (Sc), *Kit* (SG), *Spesp1* (SZ), and *Sox9* (SC). *Actb* was used as an internal control.

**Figure S3**

**Figure S3. Generation and transcriptomic profiling of *Mtch2* knockout spermatogonia.**

(A) Results of monoclonal PAGE non-denaturing gel separation in HeLa and GC-1 cell lines. The red box indicates clones with potential successful knockout.

(B) Sequence alignment of monoclonal DNA sequencing in HeLa and GC-1 cell lines. The knockout target site is located on the first exon of the *Mtch2* gene. Sequence alignment revealed that deletion mutations with non-multiples of three had occurred on both DNA strands of the clones.

(C) Representative confocal images of cell proliferation assessed by EdU staining. Scale bar, 50 µm.

(D) Western blot analysis of LC3B-II and p62 levels in sorted testicular cell populations. GAPDH was used as an internal control.

(E) Scatter plot of differentially expressed genes (DEGs) between wild-type and *Mtch2* knockout spermatogonia. Orange dots represent upregulated genes (1,371), blue dots represent downregulated genes (2,714), and black dots represent unchanged genes (fold change > 1.2 & FDR < 0.05).

(F) Hierarchical clustering of DEG expression profiles. Color intensity indicates Log_2_(FPKM) relative to the mean (scale bar). FPKM: Fragments per kilobase of transcript per million mapped fragments.

(G and H) KEGG enrichment analysis of down-regulated (G) and up-regulated (H) DEGs. The numbers represented the actual number of DEGs that were classiﬁed in a particular pathway. X-axis indicated the percentage of the DEGs in the pathway.

**Figure S4**

**Figure S4. Transcriptomic analysis reveals altered autophagy-related gene expression in *Mtch2*-deficient spermatogonia.**

(A) Heatmaps indicated the differential expression of autophagy-related DEGs between WT and *Mtch2*-KO spermatogonia.

(B) Heatmaps indicated the differential expression of mitophagy-related DEGs between WT and *Mtch2*-KO spermatogonia.

(C) Statistical analysis of the autophagy-related DEGs (FDR < 0.05) based on KEGG analysis. Red bars indicated pro-autophagy genes and purple bars represented anti-autophagy genes.

**Figure S5**

**Figure S5. MTCH2 inhibits autophagy in HeLa cells.**

(A) LC3B puncta formation in HeLa cells under EBSS-induced starvation for the indicated time and analyzed by immunoﬂuorescence with the anti-LC3B antibody (green). The nuclei were stained with DAPI reagent. Scale bar, 5 μm.

(B) Quantification of LC3B puncta from (A). Data are presented as mean ± SEM. *p < 0.05; **p < 0.01 (n = 3 independent experiments).

(C) Immunofluorescence co-localization (yellow) of MTCH2 (red) and LC3B (green) were performed with anti-MTCH2, anti-LC3B, goat anti-rabbit secondary antibody in HeLa cells. Scale bar, 5 μm.

(D) Co-localization of MTCH2 (red) and LC3B (green) in sperm mid-pieces (mitochondrial sheath). The spermatozoa were extracted from epididymis of adult mice. Scale bar, 100 μm.

**Figure S6**

**Figure S6. MTCH2 inhibits autophagy in GC-1 cells.**

(A and B) Western blot analysis of LC3B-II and p62 levels in GC-1 cells under indicated conditions. GAPDH was used as an internal control.

**Figure S7**

**Figure S7.** **Construction of Germ Cell-Specific *Mtch2* Conditional Knockout (cKO) Mouse Model.**

(A) Schematic of germ cell-specific *Mtch2* conditional knockout strategy using *Stra8*Cre.

(B) Western blot analysis confirming MTCH2 depletion in heterozygous testis and sperm lysates. Graph shows MTCH2/GAPDH ratio. Data are presented as means ± SEM. *, P < 0.05 (n=3 independent experiments).

(C) RT-qPCR validation of *Mtch2* depletion in heterozygous testes and sperm*.* Data: mean ± SEM (n=3). *Actb* was used as an internal control. ****p < 0.0001.

**Table S1.**

Primers.

| **Primer names** | **Sequence (5'-3')** |
| --- | --- |
| MTCH2-human-crispr-F | caccgtttcacgtacatgagcggct |
| MTCH2-human-crispr-R | aaacagccgctcatgtacgtgaaac |
| Mtch2-mus-crispr-F | caccgacacgtgatctggaaccggc |
| Mtch2-mus-crispr-R | aaacgccggttccagatcacgtgtc |
| Mtch2-GFP-F | gtttaaacttaagcttatggcggacgcggccagt |
| Mtch2-GFP-R | cagcatggtggcgcatggcggtagaaatgtca |
| Mtch2-psico-puro-F | cccggtgcctgacaattcgtcgagggacctaataacttcgtatagc |
| Mtch2-psico-puro-R | tcgtctttgtaatccatggtggcggccgccaccgcggtggag |
| Mtch2-puro-F | ccaccatggattacaaagacgatgacgataaggg |
| Mtch2-puro-R | acgaattgtcaggcaccgggcttg |
| Becn1-GFP-1-138-F | tgtgaggaatgcggatccaccggtcgccacatggtg |
| Becn1-GFP-1-138-R | ggatccgcattcctcacacagcgggtgatc |
| Becn1-GFP-139-353-F | ggatccaccggtcgccacatggtgagcaagg |
| Becn1-GFP-139-353-R | ctcgagatctgagtccggtagcgctagcggat |
| Becn1-GFP-139-353-F | gactcagatctcgagacagacactcttttagaccagctggacact |
| Becn1-GFP-139-353-R | tggcgaccggtggatccagaacagtataacggcaactcc |
| Becn1-GFP-354-448-F | agatctcgaggggggtttgcggtttttctgggaca |
| Becn1-GFP-354-448-R | aaacccccctcgagatctgagtccggtagcgctagc |
| Becn1-GFP-1-353-F | ttctggatccaccggtcgccacatggtgagcaa |
| Becn1-GFP-1-353-R | accggtggatccagaacagtataacggcaactcctt |
| Becn1-GFP-106-448-F | tcagatctcgagaccatggagaacctcagccgga |
| Becn1-GFP-106-448-R | tggtctcgagatctgagtccggtagcgctagcggatctga |
| Becn1-GFP-BH3-F | tgtctctcgaacagacactcttttagaccagctggaca |
| Becn1-GFP-BH3-R | agtgtctgttcgagagacaccatcctggcgag |
| Becn1-GFP-CCD-F | tgtgtgaggaatgcacaaatgccaccttccacatctggcacagcg |
| **Primer names** | **Sequence (5'-3')** |
| Becn1-GFP-CCD-R | tgtgcattcctcacacagcgggtgatccacatcg |
| Becn1-GFP-ECD-F | aatgtcttcgagtctctgacagacaaatct |
| Becn1-GFP-ECD-R | agagactcgaagacattggttttcttgagcttgt |
| Becn1-myc-Atg6-ECD-F | aaaaccaatgtcttcgagtctctgacagac |
| Becn1-myc-Atg6-ECD-R | gtctgtcagagactcgaagacattggtttt |
| Becn1-myc-Atg6-CCD-F | gcacaaatgccaccttccacatctggca |
| Becn1-myc-Atg6-CCD-R | catttgtgcattcctcacacagcgg |
| Becn1-myc-Atg6-1-353-F | tactgttcttgactcgagcatgcatctagagggccc |
| Becn1-myc-Atg6-1-353-R | ctcgagtcaagaacagtataacggcaactccttaga |
| Becn1-myc-Atg6-139-448-F | aggccgaattcacagacactcttttagaccagct |
| Becn1-myc-Atg6-139-448-R | tctgtgaattcggcctccatggccatatgcaggt |
| Becn1-myc-Atg6-1-105-F | tgatggtggctgactcgagcatgcatctagaggg |
| Becn1-myc-Atg6-1-105-R | tcgagtcagccaccatcagatgcctccccgat |
| Becn1-myc-Atg6-106-448-F | aggccgaattcaccatggagaacctcagcc |
| Becn1-myc-Atg6-106-448-R | tccatggtgaattcggcctccatggccatatgca |
| Mtch2-muscell-ko-F | acaagatcgaattctaagcgggcggcgcg |
| Mtch2-muscell-ko-R | agcgcggatcctgacaggacgcgggc |
| MTCH2-homocell-ko-F | tacaagatcgaattcttggtggctccgtctgt |
| MTCH2-homocell-ko-R | tcgagcgcggatccaggagcagcagcatct |
| Mtch2-muscell-F | ctccaacaataggacgaaat |
| Mtch2-muscell-R | taatgagggtagcagcagaa |
| Gapdh-RT-F | gcagtggcaaagtggagatt |
| Gapdh-RT-R | cgctcctggaagatggtgat |
| Sox9-F | aggtgctgaagggctacgactg |
| Sox9-R | tcggcggaccctgagattgc |
| Dazl-F | ttaggatggatgaaaccgaaat |
| Dazl-R | tctggaactgtggtggagga |
| Stra8-Cre-F | gtgcaagctgaacaacagga |
| **Primer names** | **Sequence (5'-3')** |
| Stra8-Cre-R | agggacacagcattggagtc |
| Flox1-Mtch2-mus-F | cccagagttcactttatgttacgg |
| Flox1-Mtch2-mus-R | catgttcacagaggtcgagtgca |
| Flox2-Mtch2-mus-F | gactcaactcaaagggaaagttgg |
| Flox2-Mtch2-mus-R | aaatggacccaatgtccatcctta |
| Akap3-RT-F | aattccctgagaacatccg |
| Akap3-RT-R | tgctcacccagagtttcg |
| Cfap251-RT-F | tcttgatgcgatgtcttta |
| Cfap251-RT-R | aggaggctttggatgctg |
| mcherry-GFP-Fis1-F | ctcgagctcaagcttatggaggccgtgctgaacga |
| mcherry-GFP-Fis1-R | tcgactgcagaattctcaggatttggacttggaga |
| Fis1-1 | atggaggccgtgctgaacgagctggtgtctgtggaggatctgaagaattttgaaa |
| Fis1-2 | ttggacacagaaccagctgcctgctcagactgaaatttcctttcaaaattcttca |
| Fis1-3 | tggttctgtgtccaagagcacgcaatttgaatatgcctggtgcctggttcgaagc |
| Fis1-4 | ccagcagcacgatgcctctgcggatgtcctcattgtatttgcttcgaaccaggca |
| Fis1-5 | gcatcgtgctgctggaggagctgttgcccaaagggagcaaagaggaacagcggga |
| Fis1-6 | cttgagccggtagttgcccacggccaggtagaagacatagtcccgctgttcctct |
| Fis1-7 | aactaccggctcaaggaatatgaaaaggctctaaagtatgtgcgagggctgttgc |
| Fis1-8 | cgttccagctccttggcctggttgttctggggctcagtctgcaacagccctcgca |
| Fis1-9 | caaggagctggaacgcctgattgataaggccatgaagaaagatggactggtaggc |
| Fis1-10 | gggccatgccaccaacgatggccatgcctaccagtccatc |
| Fis1-11 | ttggtggcatggccctgggcgtggcaggcctggctggact |
| Fis1-12 | tcaggatttggacttggagacagccagtccaatgagtccagccaggcct |
